# Health Economic Evaluations of Circulating Tumor DNA Testing for Cancer Screening: Systematic Review

**DOI:** 10.1002/cam4.70641

**Published:** 2025-02-05

**Authors:** Mingjun Rui, Yingcheng Wang, Joyce H. S. You

**Affiliations:** ^1^ School of Pharmacy, Faculty of Medicine The Chinese University of Hong Kong Hong Kong SAR China

**Keywords:** cancer, circulating tumor DNA, screening, systematic review

## Abstract

**Background:**

Cancer detection remains a significant global healthcare challenge, and circulating tumor DNA (ctDNA) is a biomarker for noninvasive cancer screening.

**Objective:**

This systematic review aimed to describe health economic evaluations of ctDNA for cancer screening.

**Methods:**

A comprehensive literature search was performed (following PRISMA guidelines) across MEDLINE, Embase, APA PsycINFO, Cochrane Library, Web of Science, and the Center for Review and Dissemination. The review included full‐scale health economic analyses such as cost–effectiveness, cost–utility, cost–benefit, and cost–consequence analyses. The quality of the included reports was assessed using CHEERS 2022 standards, and each report was categorized as excellent, very good, good, or insufficient.

**Results:**

Eighteen studies were selected, including four ctDNA tests (EBV‐DNA, cf‐DNA, mSEPT9, and mt‐sDNA) for three types of cancer screening: nasopharyngeal carcinoma (NPC) (2; 11.11%), breast cancer (BC) (1; 5.56%), and colorectal cancer (CRC) (15; 83.33%). Five studies (27.78%) found ctDNA cost‐effective for CRC screening (mt‐sDNA (with higher uptake than conventional tests) versus fecal immunochemical testing (FIT) or colonoscopy (*n* = 4); mSEPT9 versus computed tomography colonoscopy (CTC) (*n* = 1)). Thirteen studies (72.22%) found ctDNA not cost‐effective for NPC (EBV‐DNA versus no screening (*n* = 2)); BC (cf‐DNA versus conventional testing (*n* = 1)); CRC (mSEPT9 versus FIT or colonoscopy (*n* = 2)); mt‐sDNA versus FIT or colonoscopy (*n* = 5); mSEPT9 or mt‐sDNA versus conventional tests (*n* = 3)). The CHEERS assessment found all reports in the “very good” category.

**Conclusion:**

All ctDNA tests were generally not cost‐effective comparing to conventional screening methods, except when the mt‐sDNA uptake was higher than the comparators or when mSEPT9 was compared with CTC.

**Trial Registration:**

CRD42023477732

## 1 Introduction

Detection of cancer is a global healthcare challenge, and early detection is critical to provide timely cancer treatment [1, 2, 3]. In recent years, the application of circulating tumor DNA (ctDNA) as a biomarker for liquid biopsy has emerged for cancer screening [4, 5, 6, 7, 8]. ctDNA is a subset of cell‐free DNA (cf‐DNA) that originates from tumor cells [4, 5]. It carries tumor‐specific genetic information and circulates in the bloodstream [9]. ctDNA testing is used as a non‐invasive tool for the early detection of malignancies and has shown potentials to improve cancer manangement [10].

Health economic evaluations play a key role in informing decisions on resource allocation to new technology in clinical practice. The rapid advancement of ctDNA technology had led to the increasingly reported health economic analyses of ctDNA testing for cancer screening. A recent systematic review of 24 health economic evaluations found that blood‐based liquid biopsy was applied for cancer management in screening (66.67%), guiding treatment selection (26.67%), and treatment monitoring (6.67%) [11]. The observed findings suggested that blood‐based liquid biopsies may be cost‐effective for screening and early detection of colorectal, gastric, and brain cancers. Recently, increasing health economic studies on non‐blood‐based application of ctDNA for cancer screening are reported [12, 13, 14]. This systematic review aimed to describe health economic evaluations of blood‐based and non‐blood‐based ctDNA technology in cancer screening and evaluate the quality of the included reports.

## 2 Methods

### 2.1 Search Strategy

A comprehensive literature search strategy was developed to assess the health economic evaluations of ctDNA technology for cancer screening in undiagnosed individuals. An initial search was conducted to identify relevant search terms related to ctDNA technology, such as “liquid biopsy”, “circulating tumor DNA”, “cell‐free DNA”, and “biomarker.” Our search strategy was constructed by combining search terms encompassing “cancer”, “health economic evaluation”, and “ctDNA technology.” Based upon the choices of databases reported in prior systematic reviews on health economic evaluations of genetic testing of cancers [15, 16, 17], our search was conducted (from 2001 to October 2023) in MEDLINE (Ovid), Embase (Ovid), APA PsycINFO (Ovid), Cochrane library, Web of Science, and Center for Review and Dissemination. A manual search of the reference lists from both included studies and associated systematic reviews was performed to ensure comprehensive coverage. This systematic review has been registered with PROSPERO (registration ID: CRD42023477732). Details of the search strategies and the PRISMA (Preferred Reporting Items for Systematic Reviews and Meta‐Analyses) 2020 checklist are shown in Tables S1 and S2 [18].

### 2.2 Eligibility Criteria

The inclusion criteria were in compliance with the PICOS [19, 20]:

Patient (P): Asymptomatic or undiagnosed population.

Intervention (I): Blood‐based and non‐blood‐based cfDNA, circulating tumor DNA or methylated DNA technology.

Comparator (C): No cancer screening or non‐ctDNA‐based cancer screening.

Outcomes (O): Health economic outcomes (such as cost, life years, quality adjusted life years, and incremental cost‐effectiveness ratios) generated by full‐scale health economic analyses including cost–effectiveness analysis (CEA), cost–utility analysis (CUA), cost–benefit analysis (CBA), and cost–consequence analysis (CCA). CEA compared the costs and effectiveness of interventions and comparators with the effectiveness measured as natural health outcomes (such as life‐years gained, cases detected, deaths). CUA evaluated the costs and effectiveness of alternative strategies with effectiveness measured as quality‐adjusted life‐years (QALYs), which combines both the quality‐of‐life and life‐years gained. CBA examined the costs and outcomes, both valued in monetary terms. CCA assessed the costs and presented effects as consequences (including health, non‐health, negative, and positive effects) of the interventions and comparators [21].

Study design (S): Model‐based (including Markov model, decision tree model, or microsimulation model) or trial‐based study design.

Full‐text English‐language health economic studies meeting the inclusion criteria were included. An article was excluded if (1) all cancer testing methods examined were unrelated to the ctDNA technology; (2) the ctDNA technology was used for non‐screening purpose(s) (such as treatment selection or post‐treatment surveillance); (3) partial health economic evaluation; and (4) studies were reported in a format other than journal articles such as letters, editorials, conference abstracts, posters, comments, and thesis.

### 2.3 Study Selection

We utilized EndNote to manage all the retrieved records. Duplicates were first removed, and a two‐stage screening process was conducted by two reviewers (RM and WY) independently. During the initial stage, titles and abstracts were assessed to determine eligibility. In the second phase, full‐text reviews were conducted to confirm the eligibility of studies based on predetermined inclusion and exclusion criteria. In cases of disagreement between reviewers (RM and WY), a third reviewer (JHSY) was consulted for resolution. The entire selection process adhered to the guidelines provided in the PRISMA statement for the preferred reporting of systematic reviews and meta‐analyses [18].

### 2.4 Data Extraction

Data extraction was carried out independently by two reviewers (RM and WY) using a standardized data extraction checklist. The extracted data included: (1) general information (author, publication year, and country); (2) disease and ctDNA information (cancer type, ctDNA type, and sample source); (3) study methodology (type of health economic evaluations, study design by model‐based or trial‐based, time horizon, study perspective, screening strategies, and source of funding); (4) health economic results (base‐case cost, QALY, and incremental cost‐effectiveness ratio (ICER), sensitivity analyses, and threshold analyses).

### 2.5 Assessment of Methodological Quality

The methodological quality of all included studies in this review was assessed using the Consolidated Health Economic Evaluation Reporting Standards (CHEERS) 2022 checklist (Table S3) [22]. The CHEERS checklist comprises 28 items organized into six categories: (1) title, (2) abstract, (3) methods, (4) results, (5) discussion, and (6) other relevant information. One point was assigned to each CHEERS item when the item‐specific requirements were fully met; 0.5 points to an item when the requirements were partially satisfied; 0 points to an item when the requirements were not satisfied. The maximum score is 28 points (100.00%). Each study was subsequently classified into one of four categories: Excellent (scored 85.00% or higher); very good (scored 70.00%–84.99%); good (scored 55.00%–69.99%); insufficient (scored less than 55.00%) [11, 23]. The evaluation of all studies was carried out independently by two investigators (RM and WY), and any disagreements were resolved through discussion with a third investigator (JHSY).

### 2.6 Data Analysis and Presentation

The selection process was presented in a flowchart. The descriptive characteristics and quality assessment of selected reports were summarized and tabulated. The study‐specific assessment on each CHEERS checklist item was also reported. The ctDNA technology for cancer screening was considered cost‐effective if it was either (1) more effective and less costly than the comparator or (2) more effective at a higher cost and the ICER was lower than the willingness‐to‐pay (WTP) threshold. The ctDNA technology was considered not cost‐effective if it was either (1) less effective and more costly than the comparator (dominated by comparator), (2) the ICER of the ctDNA technology exceeded the WTP threshold, and (3) less effective and less costly than the comparator (such scenario forgone health benefits for cost savings, thus causing ethical concerns) [24, 25]. The health economic findings of ctDNA‐based screening strategy(s) in each selected report was presented.

## 3 Results

### 3.1 Search Results

**FIGURE 1:**
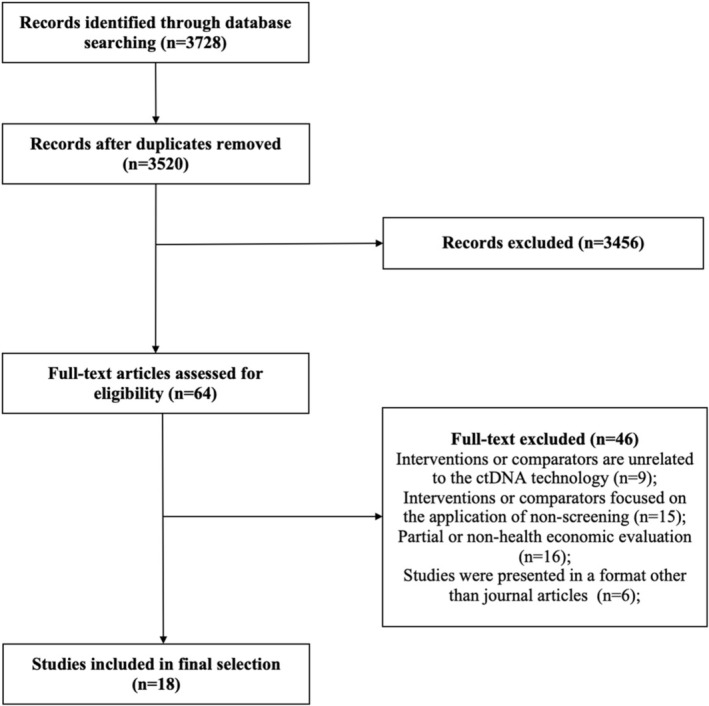
The article selection process according to the Preferred Reporting Items for Systematic Reviews and Meta‐Analyses guideline.

There were 3728 records initially identified, and 3520 reports remained after eliminating duplicates. Screening by the title and abstract further excluded 3456 reports, and full‐text evaluation was performed for 64 articles. A total of 18 studies [12, 13, 14, 26, 27, 28, 29, 30, 31, 32, 33, 34, 35, 36, 37, 38, 39, 40] were included in the final selection. The selection process is illustrated in Figure 1.

### 3.2 Methodology Characteristics

Majority of studies (17/18, 94.44%) were conducted in high‐income countries/regions, including the United States, Germany, Australia, France, and Taiwan [12, 13, 14, 26, 28, 29, 30, 31, 32, 33, 34, 35, 36, 37, 38, 39, 40]. One study (1/18, 5.56%) further covered 132 countries ranging from high‐, middle‐, and low‐income countries/regions [27]. Three types of cancer were screened: Nasopharyngeal carcinoma (NPC) (2/18, 11.11%) [26, 27], breast cancer (BC) (1/18, 5.56%) [28], and colorectal cancer (CRC) (15/18, 83.33%) [12, 13, 14, 29, 30, 31, 32, 33, 34, 35, 36, 37, 38, 39, 40]. Four different ctDNA technologies examined were plasma‐based Epstein–Barr virus‐DNA (EBV‐DNA, 2/18, 11.11%) [26, 27], plasma‐based cf‐DNA (1/18, 5.56%) [28], plasma‐based methylated Septin 9 DNA (mSEPT9, 6/18, 33.33%) [29, 30, 37, 38, 39, 40], and stool‐based multitarget stool DNA testing (mt‐sDNA, 12/18, 66.67%) [12, 13, 14, 31, 32, 33, 35, 36, 38, 39, 40]. There were 3 studies (3/18, 16.67%) that considered both mSEPT9 and mt‐sDNA [38, 39, 40]. The characteristics of 18 included studies are summarized in Table 1.

**TABLE 1:** Key characteristics of all included studies (*n* = 18).

Author & year	Country or region	Target population	Strategies evaluated	Study type; Model type; Time horizon; Cycle length; perspective; funding source	CHEERS score of 28 (%) & ranking
**EBV‐DNA for nasopharyngeal carcinoma screening (*n* = 2)**					
Harris et al. 2019 [26]	US	Asian American men aged 50 years	(1) Onetime screening using the plasma EBV‐DNA; (2) Usual care with no screening	CUA; Markov; Lifetime; 1 year; Medicare payer; Academic institution	21 (75.00%); very good
Miller et al. 2021 [27]	132 countries	Aged 50 years	(1) No screening; (2) EBV DNA plasma PCR, followed by endoscopy; (3) EBV DNA plasma PCR, followed by DNA plasma PCR surveillance, followed by endoscopy; (4) EBV DNA plasma PCR, followed by DNA plasma PCR surveillance, followed by endoscopy+ MRI nasopharynx; (5) EBV DNA plasma PCR + serum EBV VCA IgA ELISA, followed by DNA plasma PCR surveillance, followed by endoscopy; (6) Serum EBV VCA IgA ELISA + endoscopy; (7) Serum EBV VCA IgA ELISA, followed by endoscopy; (8) Serum EBNA + VCA IgA ELISA, followed by endoscopy; (9) Serum EBNA + VCA IgA ELISA‐EBV DNA plasma PCR, followed by endoscopy; (10) Serum EBNA + VCAIgA ELISA, followed by EBV EAD IgA ELISA‐endoscopy; (11) Serum EBV VCA IgA, followed by EBV DNA nasopharyngeal swab PCR, followed by endoscopy	CUA; Markov; Lifetime; 1 year; Payer; No funding	20.5 (73.21%); very good
**Circulating cell‐free DNA for breast cancer screening (*n* = 1)**					
van der Poort et al. 2022 [28]; US	US	Women aged 50–74 years	(1) Liquid biopsy screening (cf‐DNA) every 2 years; (2) Digital mammography screening every 2 years	CUA; Micro‐simulation; Lifetime; −; Federal payer; Government agencies	20.5 (73.21%); very good
**Plasma mSEPT9 for colorectal cancer screening (*n* = 3)**					
Ladabaum et al. 2013 [29]	US	Aged 50–80 years	(1) No screening; (2) mSEPT9‐2well assay every 2 years; (3) mSEPT9‐3well assay every 2 years; (4) FOBT every year; (5) SIG every 5 years; (6) FIT every year; (7) colonoscopies every 10 years; (8) SIG every 5 years/FIT every year; (9) SIG every 5 years/FOBT every year	CUA; Markov; Lifetime; 1 year; Medicare payer; Industry	20 (71.43%); very good
Ladabaum et al. 2014 [30]	Germany	Aged 50–75 years	(1) No screening; (2) colonoscopies at ages 60 and 70; (3) mSEPT9‐2well assay every 2 years; (4) FOBT every year; (5) mSEPT9‐3well assay every 2 years; (6) colonoscopies at ages 55 and 65; (7) colonoscopies/FOBT at ages 60 and 70; (8) mSEPT9‐2well assay every year; (9) mSEPT9‐3well assay every year; (10) FIT/COLO at ages 60 and 70; (11) FOBT/COLO at ages 55 and 65; (12) FIT every year	CUA; Markov; Lifetime; 1 year; Healthcare payer; Industry	20.5 (73.21%); very good
Peterse et al. 2021 [37]	US	Aged 50–75 years	(1) No screening; (2) FIT every year; (3) CTC every 5 years; (4) COLO every year; (5) mSEPT9 every 2 years; (6) mSEPT9 every year; (7) mt‐sDNA every 3 years; (8) PillCam COLON 2 every 10 years; (9) PillCam COLON 2 every 5 years; (10) mt‐sDNA every year	CUA; Micro‐simulation; Lifetime; Society; Government agency	20.5 (73.21%); very good
**mt‐sDNA for colorectal cancer screening (*n* = 9)**					
Karlitz et al. 2022 [12]	US	Aged 50–64 years	(1) No screening; (2) mt‐sDNA every 3 years; (3) outreach with FIT every year; (4) outreach without FIT every year	CUA; Micro‐simulation; Lifetime; −; Medicaid payer; Industry	22 (78.57%); very good
Redwood et al. 2021 [13]	US	Aged 40	(1) No screening; (2) mt‐sDNA every 3 years; (3) FIT every year; (4) COLO every year	CUA; Markov; Lifetime; 1 year; Health service provider; Academic institution	22.5 (79.29%); very good
Fisher et al. 2021 [14]	US	Aged 65 years	(1) No screening; (2) mt‐sDNA every 3 years; (3) FIT every year; (4) FOBT every year	CUA; Micro‐simulation; Lifetime; −; Medicare payer; Industry	22 (78.57%); very good
Hathway et al. 2020 [31]	US	Aged 50–75 years	(1) Increased mt‐sDNA scenario (28.00% mt‐sDNA+9.00% FIT+63.00% colonoscopy) (2) Status quo scenario (6.00% mt‐sDNA+11.00% FIT+83.00% colonoscopy)	CCA; Markov; 10 years; 1 year; Integrated Delivery Networks and payers; Industry	22 (78.57%); very good
Naber et al. 2019 [32]	US	Aged 65 years	(1) No screening; (2) high sensitivity guaiac‐based FOBT every year; (3) FIT every year; (4) SIG every 5 years; (5) SIG every 10 years + high sensitivity guaiac‐based FOBT every year; (6) SIG every 10 years + FIT every year; (7) COLO every 3 years or every 5 years; (8) mt‐sDNA every 3 years	CEA; Micro‐simulation; Lifetime; −; Medicare and Medicaid payer; Industry	20 (71.43%); very good
Ladabaum et al. 2016 [33]	US	Aged 50 years	(1) No screening; (2) FIT every year; (3) mt‐sDNA every 3 years; (4) COLO every year	CUA; Markov; Lifetime; 1 year; Third‐party payer; Industry	22.5 (79.29%); very good
Kingsley et al. 2016 [34]	US	Aged 50 years	(1) No screening; (2) FIT every year; (3) mt‐sDNA every 3 years; (4) COLO every year; (5) SIGB every 5 years	CUA; Markov; Lifetime; 1 year; Society; No funding	21.5 (76.79%); very good
Lansdorp‐Vogelaar et al. 2010 [35]	US	Aged 65 years	(1) No screening; (2) HII every year; (3) HS every year; (4) iFOBT every year; (5) SIGB every 5 years; (6) SIG every 5 years; (7) HII every year + SIGB every 5 years; (8) HII every year + SIG every 5 years; (9) HS every 3 years + SIGB every 5 years; (10) HS every 3 years + SIG every 5 years; (11) iFOBT every year + SIGB every 5 years; (12) iFOBTevery year + SIG every 5 years; (13) iFOBT every 3 years + SIGB every 5 years; (12) iFOBT every 3 years + SIG every 5 years; (13) COLO q 10; (14) mt‐sDNA every 3 years; (15) mt‐sDNA every 5 years	CEA; Micro‐simulation; Lifetime; −; Third‐party payer; Government agency	20.5 (73.21%); very good
Wu et al. 2006 [36]	Taiwan	Aged 50–75 years	(1) No screening; (2) mt‐sDNA every 3 years; (3) mt‐sDNA every 5 years; (4) mt‐sDNA every 10 years; (5) FOBT every year; (6) SIG every 5 years; (7) COLO every 10 years	CEA; Markov; Lifetime; 1 year; Third‐party payer; No funding	22.5 (79.29%); very good
**mSEPT9 or mt‐sDNA for colorectal cancer screening (*n* = 3)**					
Lew et al. 2018 [38]	Australia	Aged 50–74 years	No screening; (2) iFOBT every 2 years; (3) iFOBT every year; (4) mSEPT9 every 2 years; (5) mt‐sDNA every 5 years; (6) COLO every 10 years; (7) SIG every 10 years; (8) CTC Every 10 years; (9) Once‐off SIG; (10) Once‐off SIG combined with iFOBT every 2 years; (11) Once‐off COLO combined with iFOBT every 2 years; (12) iFOBT every 2 years combined with SIG for negative iFOBT; (13) iFOBT every 2 years combined with SIG for negative iFOBT; (14) iFOBT every 2 years combined with mSEPT9 DNA in under‐screened individuals	CEA; Micro‐simulation; Lifetime; Health service provider; Academic institution	22 (78.57%); very good
Benamouzig et al. 2021 [39]	France	Aged 45 years with familial history of CRC	(1) No screening; (2) FIT every 5 years; (3) CTC every 5 years; (4) COLO every 5 years; (5) mSEPT9 every 5 years; (6) mt‐sDNA every 5 years; (7) colon capsule every 5 years; (8) SIG every 5 years; (9) one time COLO + FIT every 2 years	CUA; Micro‐simulation; Lifetime; Society; Government agency	21 (75.00%); very good
Barré et al. 2020 [40]	France	Aged 50 years	(1) No screening; (2) FIT every 2 years; (3) CTC every 10 years; (4) COLO every 3 years or every 5 years; (5) mSEPT9 every 2 years; (6) mt‐sDNA every 2 years; (7) colon capsule every 10 years; (8) SIG every 10 years	CUA; Micro‐simulation; Lifetime; Society; Government agency	20 (71.43%); very good

Abbreviations: CCA, cost–consequence analysis; CEA, cost–effectiveness analysis; cf‐DNA, cell‐free DNA; COLO, colonoscopy; CTC, computed tomography colonography; CUA, cost‐utility analysis; EBNA, Epstein–Barr nuclear antigen; EBV, Epstein–Barr virus; ELISA, enzyme‐linked immunosorbent assay; FIT/COLO, hybrid strategies with FIT and colonoscopy; FIT, fecal immunochemical testing; FOBT/COLO, hybrid strategies with FOBT and colonoscopy; FOBT, fecal occult blood testing; HII, Hemoccult II; HS, Hemoccult SENSA; IDNs, Integrated Delivery Networks; IFOBT, immunochemical fecal occult blood test; IgA, immunoglobulin A; MRI, magnetic resonance imaging; MSEPT9, methylated Septin 9 DNA (mSEPT9‐2‐well assay used 2 polymerase chain reaction (PCR) wells to test for mSEPT9, mSEPT9‐3‐well used 3 PCR wells to test for mSEPT9); MT‐SDNA, multitarget stool DNA testing; PCR, polymerase chain reaction; SIG, sigmoidoscopy; SIGB, sigmoidoscopy with biopsy; VCA, viral capsid antigen.

All 18 included reports were model‐based analyses: 13 studies were cost–utility analyses (72.22%) [12, 13, 14, 26, 27, 28, 29, 30, 33, 34, 37, 39, 40], 4 (22.22%) were cost–effectiveness analyses [32, 35, 36, 38], and one (5.56%) was CCA [31]. The perspectives of health economic analysis included provider (10/18, 55.56%) [12, 13, 14, 26, 28, 29, 30, 31, 32, 38], third‐party payer (4/18, 22.22%) [27, 33, 35, 36], and society (4/18, 22.22%) [34, 37, 39, 40]. The funding sources of included reports were industry (7/18, 38.89%) [12, 14, 29, 30, 31, 33], government agencies (5/18, 27.78%) [28, 35, 37, 39, 40], and academic institutions (3/18, 16.67%) [13, 26, 38]. Three studies received no funding [27, 34, 36].

Nine (50.00%) of 18 included studies used yearly‐cycle Markov models [13, 26, 27, 29, 30, 31, 33, 34, 36] and 9 (50.00%) used microsimulation models [12, 14, 28, 32, 35, 37, 38, 39, 40]. Life‐long timeframe was applied in 17 studies (94.44%) [12, 13, 14, 26, 27, 28, 29, 30, 32, 33, 34, 35, 36, 37, 38, 39, 40], and 10‐year time horizon was used in one study (5.56%) [31]. All models included the key health states of the target cancer type to simulate the outcomes of undetected and detected cancer over an adequate period of time.

### 3.3 Study Quality

The completeness of economic evaluation studies was assessed using the updated CHEERS checklist (2022), and the total score of each included reported is showed in Table 1. The mean CHEERS score was 21.25 (SD 0.97). There were 0 (0.00%), 18 (100.00%), 0 (0.00%), and 0 (0.00%) studies classified as excellent, very good, good, and insufficient, respectively. Four items were not fulfilled by most studies (> 50.00%): (1) Health economic analysis plan, (2) characterizing distributional effects, (3) approach to engagement with patients and others affected by the study, (4) effect of engagement with patients and others affected by the study. The itemized scores for each study were shown in Table S4.

### 3.4 Health Economic Findings of ctDNA

**FIGURE 2:**
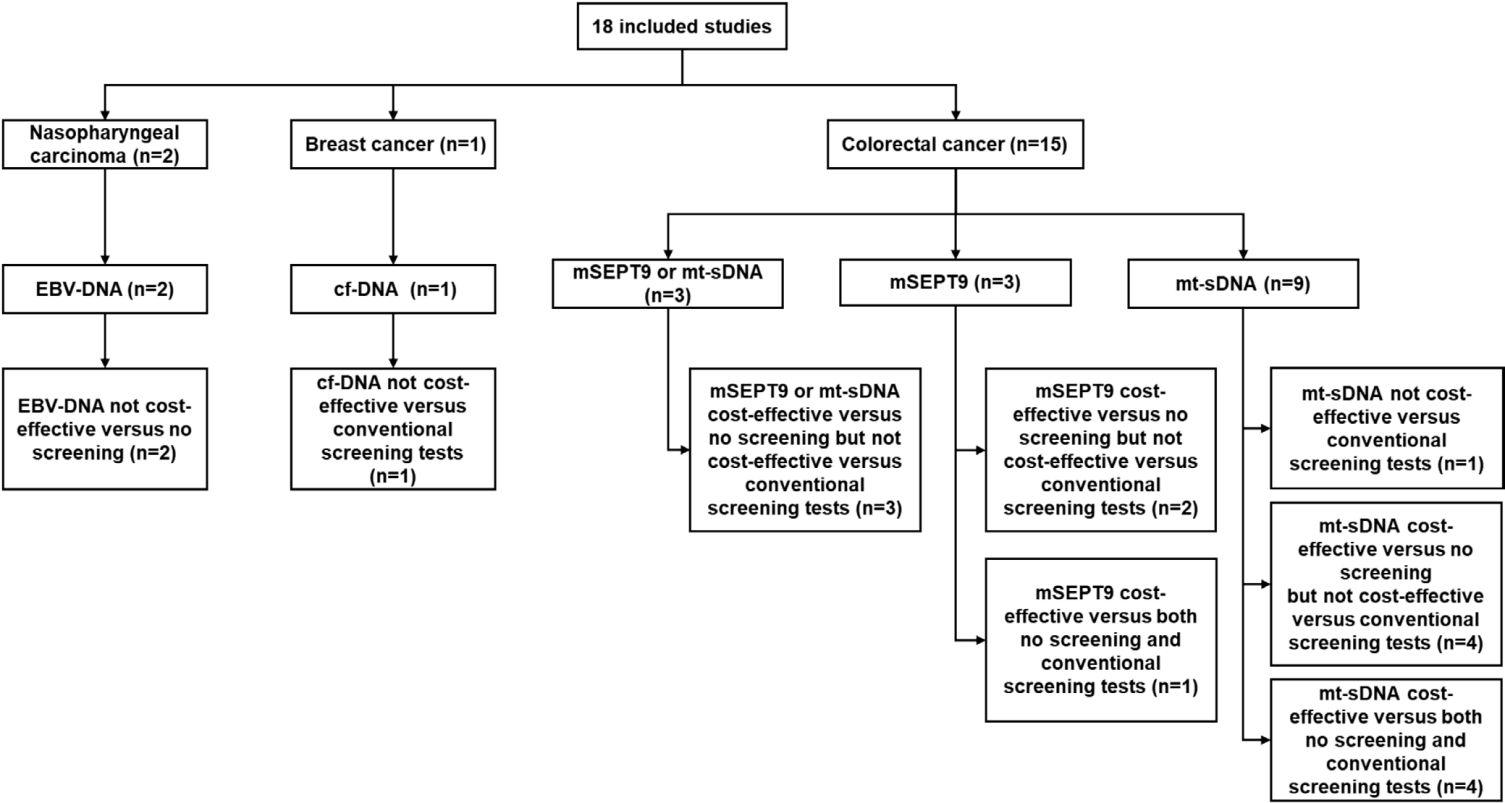
Summary of health economic evaluation results of ctDNA‐based tests for cancer screening in 18 included studies.

Comparing to “no screening”, 14 of 18 included studies (77.78%) indicated that ctDNA screening was cost‐effective [12, 13, 14, 29, 30, 32, 33, 34, 35, 36, 37, 38, 39, 40], and 2 (11.11%) studies found ctDNA screening not cost‐effective [26, 27]. Two (11.11%) studies did not consider “no screening” as an option [28, 31]. When compared to conventional screening strategies, 5 of 18 studies (27.78%) (Table 2) reported ctDNA screening to be cost‐effective [12, 13, 14, 31, 37], and all were screening for CRC. There were 11 studies (61.11%) found ctDNA screening not cost‐effective comparing to conventional testing [28, 29, 30, 32, 33, 34, 35, 36, 38, 39, 40]. Two studies (11.11%) did not consider conventional screening methods as the comparators [26, 27]. The 13 studies (72.22%) found ctDNA technologies not cost‐effective comparing to conventional testing are summarized in Table 3. All included studies (100.00%) conducted sensitivity analyses: Probabilistic sensitivity analysis in 13/18 studies (72.22%) [12, 13, 14, 26, 27, 29, 30, 31, 33, 34, 37, 39, 40], and deterministic sensitivity analysis in 16/18 (88.89%) reports [12, 13, 14, 26, 27, 28, 29, 30, 31, 32, 33, 34, 35, 36, 38, 40]. The health economic evaluations results of ctDNA tests for cancer screening in the 18 included studies are summarized in the Figure 2.

**TABLE 2:** Summary of outcomes of studies reported ctDNA to be cost‐effective for colorectal cancer screening (versus conventional strategies) (*n* = 5).

Author & year	Intervention	Comparator	Currency year	ICER versus comparator	ICER vs. “no screening”	WTP threshold	Cost‐effective probability of ctDNA in probabilistic sensitivity analysis	Influential factors and threshold values (if any) in deterministic sensitivity analysis
Karlitz et al. 2022 [12]	mt‐sDNA every 3 years	FIT every year	2021 US$	US$32,150/QALY	US$11,272/QALY	US$100,000/QALY	100.00%	Adherence had the largest impact on the ICER. mt‐sDNA every 3 years was cost‐effective unless the adherence of FIT every year exceeded 40.00%–85.00%
Redwood et al. 2021 [13]	mt‐sDNA every 3 years	FIT every year	2018 US$	US$32,047/QALY	US$31,341/QALY	US$50,000/QALY‐US$100,000/QALY	100.00%	Costs related to colonoscopy, mt‐sDNA, FIT and treatment and performance of MT‐sDNA and FIT had the largest impact on the ICER, with no threshold value identified.
Fisher et al. 2021 [14]	mt‐sDNA every 3 years	FIT every year	2020 US$	US$62,814/QALY	US$5100/QALY	US$100,000/QALY	100.00%	Adherence to follow‐up colonoscopy for mt‐sDNA had the largest impact on the ICER, with no threshold value identified.
Hathway et al. 2020 [31]	mt‐sDNA every 3 years	COLO every 10 years	2019 US$	More effective and less costly (Incremental cost: US$(4.4–19.6) Incremental effectiveness: 5 cases of CRC detected	“No screen” not an option	—	98.40%–100.00%	mt‐sDNA cost had the largest impact on the ICER, with no threshold value identified.
Peterse et al. 2021 [37]	mSEPT9 every year	CTC every 5 years	2017 US$	US$63,253/QALY	US$6639/QALY	US$100,000/QALY	54.00%	Not reported

Abbreviations: COLO, colonoscopy; CTC, computed tomography colonography; FIT, fecal immunochemical testing; mSEPT9, methylated Septin 9 DNA; mt‐sDNA, multitarget stool DNA testing.

**TABLE 3:** Summary of outcomes of studies reported ctDNA to be not cost‐effective (versus conventional testing or no screening) (*n* = 13).

Author & year	Intervention	Comparator	Currency year	ICER vs. comparator	ICER vs. “no screening”	WTP threshold	Cost‐effective probability of ctDNA in probabilistic sensitivity analysis	Influential factors and threshold values (if any) in deterministic sensitivity analysis
**ctDNA (EBV‐DNA) to be not cost‐effective vs. no screening for nasopharyngeal carcinoma screening (*n* = 2)**								
Harris et al. 2019 [26]	One‐time EBV‐DNA for nasopharyngeal carcinoma screening	Usual care without screening	2017 US$	US$113,341/QALY	US$113,341/QALY	US$100,000/QALY	35.00%	One‐time EBV‐DNA would be cost‐effective when EBV‐DNA specificity > 0.99 and prevalence of undiagnosed NPC > 40 per 100,000 and EBV‐DNA cost < $39
Miller et al. 2021 [27]	One‐time EBV‐DNA	No screening	2019 US$	US$13.48–19.33/QALY/GDP	US$13.48–19.33/QALY/GDP	US$2/QALY/GDP	5.30%–6.10%	Screening costs and discount rate had the largest impact on the ICER, with no threshold value identified.
**ctDNA (cfDNA) to be not cost‐effective vs. conventional strategies for breast cancer screening (*n* = 1)**								
Van der Poort et al. 2022 [28]	cf‐DNA every 2 years for breast cancer screening	Digital mammography every 2 years	2020 US$	>US$50,000/QALY	“No screen” not an option	US$50,000/QALY	Not reported	cf‐DNA every 2 years would be cost‐effective when cf‐DNA cost < US$187
**ctDNA (mSEPT9) to be not cost‐effective vs. conventional strategies for colorectal cancer screening (*n* = 2)**								
Ladabaum et al. 2013 [29]	mSEPT9‐2well assay every 2 years	FIT every year	2010 US$	Dominated (Incremental cost: US$1117; Incremental effectiveness: −0.02310 QALYs)	US$11,500/QALY	US$50,000/QALY	0.00%	Costs of colonoscopy, colorectal cancer care, and colorectal cancer risk had the largest impact on the ICER, with no threshold value identified.
	mSEPT9‐3well assay every 2 years	FIT every year	2010 US$	Dominated (Incremental cost: US$1018; Incremental effectiveness: −0.01510 QALYs)	US$8400/QALY	US$50,000/QALY	0.00%	
Ladabaum et al. 2014 [30]	mSEPT9‐2well assay every 2 years	FIT every year/COLO every 10 years	2011 €	Dominated (Incremental cost: €1235; Incremental effectiveness: −0.03410 QALYs)	€600/QALY	€25,000/QALY	0.00%	Test performance characteristics and costs, cancer care costs and complication rates had the largest impact on the ICER. mSEPT9‐2well or 3well assay would be cost‐effective when the test sensitivity was 15.00% for Small polyp, 19.00% for large polyp, 75.00% for localized cancer, and 100.00% for regional cancer and the test specificity was 90.00% and the cost of mSEPT9‐3 < €30
	mSEPT9‐3well assay every 2 years	FIT every year/COLO every 10 years	2011 €	Dominated (Incremental cost: €1025; Incremental effectiveness: −0.02270 QALYs)	More effective and less costly (Incremental cost: €165; Incremental effectiveness: −0.083 QALYs)	€25,000/QALY	0.00%	
	mSEPT9‐2well assay every year	FIT every year/COLO every 10 years	2011 €	Dominated €1541; Incremental effectiveness: −0.01400 QALYs	€3600/QALY	€25,000/QALY	0.00%	
	mSEPT9‐3well assay every year	FIT every year/COLO every 10 years	2011 €	Dominated (Incremental cost: €1289; Incremental effectiveness: −0.00750 QALYs)	€800/QALY	€25,000/QALY	0.00%	
**ctDNA (mt‐sDNA) to be not cost‐effective vs. conventional strategies for colorectal cancer screening (*n* = 5)**								
Naber et al. 2019 [32]	mt‐sDNA every 3 years	COLO every 10 years	2017 US$	Dominated (Incremental cost: US$1408; Incremental effectiveness: −0.0281 LYs)	More effective and less costly (Incremental cost: US$41; Incremental effectiveness: 0.079 LYs)	US$100,000/LY	Not reported	mt‐sDNA every 3 years would be cost‐effective when mt‐sDNA cost < $18 or adherence with mt‐sDNA was 31.00%–53.00% better than COLO every 10 years
Ladabaum et al. 2016 [33]	mt‐sDNA every 3 years	FIT every year	2015 US$	Dominated (Incremental cost: US$2783; Incremental effectiveness: −0.0047 QALYs)	US$27,500/QALY	US$100,000/QALY	0.00%	mt‐sDNA would be cost‐effective if used for one‐time testing instead of testing every 3 years, or if the patient support cost per FIT testing was $242.
Kingsley et al. 2016 [34]	mt‐sDNA every 3 years	FIT every year	2014 US$	US$1,391,228/QALY	US$15,762/QALY	US$50,000/QALY	0.00%	mt‐sDNA every 3 years would be cost‐effective when mt‐sDNA cost < $75
Lansdorp‐Vogelaar et al. 2010 [35]	mt‐sDNA every 3 years	COLO every 10 years	2007 US$	Dominated (Incremental cost: US$882; Incremental effectiveness: −0.0213 LYs)	US$15,285/LY	US$100,000/LY	Not reported	mt‐sDNA every 3 years would be cost‐effective when cost < $60
	mt‐sDNA every 5 years	COLO every 10 years	2007 US$	Dominated (Incremental cost: US$615; Incremental effectiveness: −0.0341 LYs)	US$12,238/LY	US$100,000/LY	Not reported	
Wu et al. 2006 [36]	mt‐sDNA every 3 years	COLO every 10 years	2004 US$	Dominated (Incremental cost: US$13,794; Incremental effectiveness: −0.01140 LYs)	US$13,615/LY	US$13,000/LY	Not reported	If the cost per mt‐sDNA test exceeds $57.1, the referral rate for diagnostic colonoscopy falls below 67.00%, or the prevalence of large adenoma at age 50 is less than 2.42%, then mt‐sDNA testing every 3 years would not be cost‐effective compared to no screening
	mt‐sDNA every 5 years	COLO every 10 years	2004 US$	Dominated (Incremental cost: US$9234; Incremental effectiveness: −0.01560 LYs)	US$9054/LY	US$13,000/LY	Not reported	
	mt‐sDNA every 10 years	COLO every 10 years	2004 US$	Dominated (Incremental cost: US$5013; Incremental effectiveness: −0.01900 LYs)	US$4834/LY	US$13,000/LY	Not reported	
**ctDNA (mSEPT9 or mt‐sDNA) to be not cost‐effective vs. conventional strategies for colorectal cancer screening (*n* = 3)**								
Lew et al. 2018 [38]	mSEPT9 every 5 years	iFOBT every 2 years	2015 AU$	Dominated (Incremental cost: AU$1266; Incremental effectiveness: −0.00310 LYs)	AU$42,710/LY	AU$50,000/LY	Not reported	Costs of colonoscopy and cancer treatment had the largest impact on the ICER, with no threshold value identified.
	mt‐sDNA every 5 years	iFOBT every 2 years	2015 AU$	Dominated (Incremental cost: AU$1494; Incremental effectiveness: −0.00200 LYs)	AU$47,727/LY	AU$50,000/LY	Not reported	
Benamouzig et al. 2021 [39]	mSEPT9 every 5 years	SIG every 5 years	2018 €	Dominated (Incremental cost: €543; Incremental effectiveness: −0.00095 QALYs)	€15,498/QALY	€40,000/QALY	0.00%	Not reported
	mt‐sDNA every 5 years	SIG every 5 years	2018 €	Dominated (Incremental cost: €123; Incremental effectiveness: −0.00603 QALYs)	€11,623/QALY	€40,000/QALY	0.00%	
Barré et al. 2020 [40]	mSEPT9 every 2 years	FIT every 2 years	2018 €	€154,600/QALY	€154,621/QALY	€40,000/QALY	0.00%	Test performance characteristics of FIT had the largest impact on the ICER, with no threshold value identified.
	mt‐sDNA every 2 years	FIT every 2 years	2018 €	€2,852,100/QALY	€23,810/QALY	€40,000/QALY	3.70%	

Abbreviations: cf‐DNA, cell‐free DNA; COLO, colonoscopy; CTC, computed tomography colonography; EBV, Epstein–Barr virus; FIT/COLO, hybrid strategies with FIT and colonoscopy; FIT, fecal immunochemical testing; IFOBT, immunochemical fecal occult blood test; MSEPT9, methylated Septin 9 DNA (mSEPT9‐2‐well assay used 2 polymerase chain reaction (PCR) wells to test for mSEPT9, mSEPT9‐3‐well used 3 PCR wells to test for mSEPT9); MT‐SDNA, multitarget stool DNA testing; PCR: polymerase chain reaction; SIG, sigmoidoscopy.

### 3.5 Type of ctDNA Testing for Cancer Screening

#### 3.5.1 EBV‐DNA for NPC Screening

Two studies evaluated EBV‐DNA for NPC screening [26, 27]. One study found that the plasma EBV‐DNA testing gained incremental QALYs at higher cost versus no screening, with an ICER of US$113,341/QALY (exceeded the WTP threshold US$100,000 per QALY) [26]. Another study reported that plasma EBV‐DNA (as the initial step of screening strategy) did not demonstrate cost‐effectiveness when compared to no screening test, and the ICER per GDP (US$13‐19/QALY/GDP) exceeded the WTP threshold of US$2/QALY/GDP. The probability of EBV‐DNA screenings being cost‐effective was 5.30%–6.10% [27].

#### 3.5.2 Cf‐DNA for BC Screening

One US study compared cf‐DNA versus digital mammography for BC screening in women aged 50–74 years and found that cf‐DNA was cost‐effective with ICER<US$50,000/QALY when the price per test was < US$187 [28].

#### 3.5.3 mSEPT9 for CRC Screening

mSEPT9 is a blood‐based biomarker of colorectal neoplasia. Six studies evaluated mSEPT9 for CRC screening, and all (100.00%) found that mSEPT9 was cost‐effective when compared with no screening [29, 30, 37, 38, 39, 40]. Comparing to conventional screening methods, four of six (66.67%) studies reported that mSEPT9 increased cost and gained lower QALYs (or life‐years) and was dominated by conventional methods [29, 30, 38, 39]. Two (33.33%) studies showed that mSEPT9 increased QALYs at higher cost when compared to conventional methods [37, 40]. One of these two studies reported that mSEPT9 was cost‐effective versus computed tomography colonography (CTC), and the ICER was US$63,253/QALY (lower than WTP threshold US$100,000/QALY) [37], and another study found that mSEPT9 (ICER €154,600/QALY) was not cost‐effective comparing to fecal immunochemical testing (FIT) at a WTP threshold of €40,000/QALY [40].

#### 3.5.4 Mt‐sDNA for CRC Screening

mt‐sDNA is a stool‐based test comprised of DNA and hemoglobin markers. Twelve studies examined mt‐sDNA screening for CRC screening, and all (100.00%) found that the mt‐sDNA was cost‐effective versus no screening [12, 13, 14, 31, 32, 33, 34, 35, 36, 38, 39, 40]. When comparing to conventional screening tests, 6 of 12 (50.00%) studies reported that mt‐sDNA was less effective but more costly [32, 33, 35, 36, 38, 39], five (41.67%) found mt‐sDNA to gain incremental QALYs at higher cost [12, 13, 14, 34, 40], and one (8.33%) showed cost‐saving [31]. Of the 5 studies reporting increased QALYs and costs by mt‐sDNA, the ICERs of mt‐sDNA (US$32,047/QALY—US$62,814/QALY) were lower than the WTP threshold (US$100,000/QALY) in 3 studies [12, 13, 14], and the ICERs (US$1,391,228/QALY [34], €2,852,100/QALY [40]) exceeded WTP threshold in 2 other reports.

## 4 Discussion

This is the first systematic review focused on the health economic outcomes of ctDNA technologies for cancer screening. After a comprehensive searching, the review identified 18 studies evaluating the health economics of four different ctDNA technologies (plasma‐based EBV‐DNA, plasma‐based cf‐DNA, plasma‐based mSEPT9, and stool‐based mt‐sDNA) in three types of cancer screening (NPC, BC, and CRC). Of the cancer diseases targeted by the screening strategies, CRC was the most frequently studied (15/18, 83.33%) [12, 13, 14, 29, 30, 31, 32, 33, 34, 35, 36, 37, 38, 39, 40]. The quality of all the included studies were ranked “very good” using the CHEERS checklist. Most included studies (17/18, 94.44%) were conducted in high‐income countries/regions [12, 13, 14, 26, 28, 29, 30, 31, 32, 33, 34, 35, 36, 37, 38, 39, 40], and one (1/18, 5.56%) covered 132 countries ranging from high‐, middle‐, and low‐income countries/regions [27], suggesting that ctDNA technology might be more ready to be applied in developed regions. Most of the studies received funding from industry (6/18, 33.33%) [12, 14, 29, 30, 31, 33], followed by from government agencies (5/18, 27.78%) [28, 35, 37, 39, 40], and academic institutions (3/18, 16.67%) [13, 26, 38], showing that both the public sector and the technology industry have a strong interest in implementing cost‐effective ctDNA technology for cancer screening.

The included studies applied one of two model types: Markov models [13, 26, 27, 29, 30, 31, 33, 34, 36] and microsimulation models [12, 14, 28, 32, 35, 37, 38, 39, 40]. Markov modeling is characterized by the use of discrete health states to represent disease progression over fixed time intervals (such as yearly cycles) to capture long‐term outcomes [41]. Microsimulation modeling simulates individual trajectories, allowing the incorporation of heterogeneity in patient characteristics, behaviors, and disease progression [42]. Both types of modeling are widely accepted in health economic analyses, and the implications for the model findings depend on the values selected for the model input parameters [43].

Based upon the findings of the 18 included studies, the present review found that the cost‐effectiveness of ctDNA technologies for cancer screening was sensitive to the prevalence of cancer, sensitivity and specificity, and cost of ctDNA technologies. The EBV‐DNA‐based screening for NPC was deemed not cost‐effective at low NPC incidence rate, as the benefits of detecting new cases did not outweigh the cost of EBV‐DNA screening [26, 27]. The mt‐sDNA was found to be cost‐effective for CRC screening when the analysis was focused on those populations at higher risk for CRC (e.g., Alaska population) by early detection and treatment, thus resulted in CRC cases reduction [13]. Ten included studies found that the sensitivity and specificity of the tests had impact on the cost‐effectiveness of screening [26, 27, 28, 30, 31, 32, 33, 34, 39, 40]. Screening by serum EBV VCA IgA demonstrated superior cost‐effectiveness over plasma EBV‐DNA due to its higher specificity, which minimized subsequent costs associated with nasopharyngeal endoscopies [27]. Plasma mSEPT9‐based screening was not cost‐effective for CRC in most scenarios, as its performance metrics (including sensitivity and specificity) did not exhibit significant improvement over conventional screening methods [29, 30, 38, 39, 40]. Eight studies identified the cost of testing as a critical factor influencing the cost‐effectiveness of ctDNA screening [26, 27, 28, 30, 32, 34, 38]. Six of them deduced the value‐based price per test necessary for ctDNA to become cost‐effective (versus conventional screening methods) using the predetermined WTP threshold: < US$39 per test of EBV‐DNA for NPC screening [26], < US$187 per test of cf‐DNA for BC screening [28], < €30 per test of mSETP9 for CRC screening [30], < US$75 per test of mt‐sDNA for CRC screening [32, 34, 35]. Compared to the conventional screening methods, the current ctDNA technology has the characteristics of a high adherence rate, high sensitivity but low specificity [12, 13, 14, 26, 29, 30, 31, 32, 33, 34, 35, 36, 37, 38, 39, 40]. Indeed, low specificity combined with a high participation rate increased the number of false‐positive patients to undergo unnecessary diagnostic procedures after the ctDNA‐based screening.

Mixed cost‐effectiveness results of ctDNA versus traditional methods for CRC screening were observed in the included studies [12, 13, 14, 31, 32, 33, 34, 35, 36, 38, 39, 40]. The mixed results were attributed to the higher adherence to ctDNA testing than to the comparators (adopted from real‐world adherence scenarios). The studies which found ctDNA not cost‐effective for CRC screening had assumed equal adherence to all testing strategies [32, 33, 34, 35, 36, 38, 39, 40]. In the four studies which reported mt‐sDNA to be cost‐effective, the adherence rates ranged from 51.30% to 88.00% in the mt‐sDNA groups and ranged from 21.10% to 65.00% in the comparator groups [12, 13, 14, 31]. Improving the adherence to mt‐sDNA testing increased the early detection rate of CRC, thus improved the progression‐free survival and overall survival by timely interventions, and therefore enhanced the cost‐effectiveness of mt‐sDNA for CRC screening. One important direction for optimizing cost‐effectiveness of mt‐sDNA for CRC screening is to focus on increasing the population coverage rate. In addition, the selection of comparator also affected the cost‐effectiveness results. One study which reported mSEPT9 to be cost‐effective had selected the CTC every 5 years as the comparator [37]. This study did not include FIT or colonoscopy as a comparator, and both tests were considered highly cost‐effective strategies in the literature of CRC screening [29, 30, 38, 39, 40]. Those studies which evaluated mSEPT9 and FIT (or colonoscopy) as screening tests did not find mSEPT9 to be cost‐effective [29, 30, 38, 39, 40]. Notably, the adherence to colonoscopy following a positive screening test likely influences the cost‐effectiveness of the CRC screening test. Most studies applied a high (78.40%–100.00%) and the same adherence to follow‐up colonoscopy in both interventions and comparators [12, 14, 31, 32, 33, 35, 36, 38, 39, 40]. Only one of the few studies which reported mt‐sDNA to be cost‐effective for CRC screening had applied a higher adherence to follow‐up colonoscopy (87.00%–96.00%) in the mt‐sDNA group than in the comparators (22.00%–89.00%) [13]. However, none of the included studies explicitly varied both the adherence rate to ctDNA screening and adherence rate to follow‐up colonoscopy to identify the optimal combination for cost‐effective CRC screening and follow‐up. Future research on interaction of the high population coverage and adequate follow‐up colonoscopy to yield the best cost‐effective outcomes is highly warranted to provide practical guidance for healthcare implementation of ctDNA‐based CRC screening.

The findings of present systematic review suggested that ctDNA testing seems to be potentially cost‐effective for CRC screening in real‐world adherence scenarios. To facilitate the implementation of ctDNA‐based CRC screening in real‐world clinical settings, clinical trials on ctDNA testing (including sensitivity and specificity) validity, feasibility and adherence across diverse patient populations, and healthcare settings for CRC screening are warranted. Adequate clinical evidence supporting ctDNA testing is essential for adaptation of this technology in the clinical practice of CRC screening. Lowering the production cost thus, the price per test is anticipated to improve the affordability of ctDNA testing. The implementation of ctDNA‐based CRC screening is also largely affected by the uptake in the target population. Personalized reminder systems (via phone calls or SMS), offering financial incentives, and engaging healthcare providers to recommend screening during routine visits are effective measures to increase CRC screening coverage [44, 45, 46]. Health economic evaluations further focusing on ctDNA testing for CRC screening in high‐risk populations and regions with a high CRC burden are warranted to provide cost‐effectiveness information to assist policy decision‐maker in the process of resource allocation.

### 4.1 Limitations

While this systematic review conducted a comprehensive search across various databases and adhered to the PRISMA guidelines, there are several limitations in this review. The search strategy was limited to studies published in English and might miss relevant research work published in other languages. The model structures and outcome measurements varied in the included studies, and therefore limited the comparison of outcomes across these studies. The included studies were conducted in diverse healthcare settings, and the generalizability of the results to different healthcare systems, cultural contexts, and populations should be considered with caution.

Several gaps in the included studies are warranted for future research. Majority of studies were conducted in high‐income countries or regions, and health economic evaluations of ctDNA‐based cancer screening in low‐to‐middle income countries are scarce. The impact of variation in adherence to cancer screening and follow‐up confirmatory procedures (such as colonoscopy for positive CRC screening) on the cost‐effectiveness of screening strategies is yet to be addressed. The included studies examined screening strategies of three types of cancer (CRC, BC, and NPC). There are ctDNA‐based tests for screening of lung cancer, prostate cancer, melanoma [47, 48, 49], and future health economic evaluations on ctDNA‐based screening strategies for these types of cancer are essential to provide information to maximize the health benefits of the cancer screening programs.

## 5 Conclusions

This systematic review suggests that all ctDNA tests were generally not cost‐effective comparing to conventional screening methods for cancer screening. The few studies supporting the cost‐effectiveness of ctDNA (blood‐based mSEPT9 (versus CTC) and stool‐based mt‐sDNA (with higher uptake than comparators)) technologies were reported for CRC screening. The robustness of cost‐effectiveness findings was subject to the performance of the screening tests, cancer incidence rate in the target population, testing cost for cancer screening, choice of comparators, and uptake of the ctDNA‐based screening tests.

## Author Contributions

**Mingjun Rui:** conceptualization, writing – original draft, writing – review and editing, methodology, formal analysis, software, project administration, investigation. **Yingcheng Wang:** formal analysis, project administration, software, writing – original draft, investigation. **Joyce H. S. You:** conceptualization, methodology, formal analysis, supervision, writing – review and editing, investigation.

## Ethics Statement

The authors have nothing to report.

## Conflicts of Interest

The authors declare no conflicts of interest.

## Supporting information


Data S1.

## Data Availability

The authors confirm that the data supporting the findings of this study are available within the article [and/or] its Supporting Information.
