# Supplementary material for: Health Economic Evaluations of Circulating Tumor DNA Testing for Cancer Screening: Systematic Review
Source: Cancer Med. 2025 Feb 5;14(3):e70641. doi: 10.1002/cam4.70641 (PMC11795416; doi:10.1002/cam4.70641)
Supplement: Supplementary file 1 — Data S1. [file CAM4-14-e70641-s001.docx]

**Table S1 Searching Strategy of the review in different database**

| **Step** | **Query** | **Results** |
| --- | --- | --- |
| **Embase; Ovid MEDLINE(R); APA PsycInfo** | | |
| 1 | exp Neoplasms/ or (cancer* or tumo?r* or neoplas* or carcinoma*).mp. | 11943089 |
| 2 | Neoplastic Cells, Circulating/ or exp Biomarkers, Pharmacological/ or exp Biomarkers/ or exp Biomarkers, Tumor/ | 1720627 |
| 3 | (((liquid* adj3 biops*) or (plasma adj3 biops*) or (plasma adj3 DNA) or (urine adj3 DNA) or (cerebrospinal fluid adj3 DNA) or (pleural adj3 DNA) or (peritoneal fluid adj3 DNA) or (saliva adj3 DNA) or (stool adj3 DNA) or circulat* tumo?r cell* or (circulat* adj3 (cell* or tumo?r* or neoplas*)) or ctdna or cell free nucleic acid or circulating cell free rna or ccfrna or circulating cell free dna or ccfdna or biolog* marker* or biomarker* or ((cancer* or neoplasm*) adj3 blood adj3 test*) or (blood adj3 test* adj3 (cancer* or neoplasm* or carcinoma*)) or (cancer* or neoplasm* or carcinoma*)) adj3 (blood adj3 test*)).mp. | 1455 |
| 4 | 2 or 3 | 1721363 |
| 5 | 1 and 4 | 891737 |
| 6 | exp economics/ or exp cost-effectiveness/ or exp Technology Assessment, Biomedical/ | 1238356 |
| 7 | ((cost* adj3 analys*) or cost effective* or ((health* or boimed*) adj2 technolog* adj3 asses*) or (economic* adj3 evaluat*) or (health* adj2 outcom*) or (health* adj3 economic*) or (economic* adj3 analys*) or cost saving).mp. | 1131309 |
| 8 | 6 or 7 | 1944424 |
| 9 | 5 and 8 | 10461 |
| 10 | limit 9 to English language | 10161 |
| 11 | limit 10 to humans | 9687 |
| 12 | limit 11 to "review articles" | 6730 |
| 13 | 11 not 12 | 2957 |
| 14 | limit 13 to yr="2001 -Current" | 2676 |
| **Cochrane library search strategy** | | |
| #1 | MeSH descriptor: [Neoplasms] explode all trees | 112875 |
| #2 | cancer OR tumo?r OR neoplas* OR carcinoma* | 269937 |
| #3 | #1 OR #2 | 279935 |
| #4 | MeSH descriptor: [Liquid Biopsy] explode all trees | 33 |
| #5 | MeSH descriptor: [Neoplastic Cells, Circulating] explode all trees | 218 |
| #6 | MeSH descriptor: [Biomarkers, Tumor] explode all trees | 6521 |
| #7 | MeSH descriptor: [Circulating Tumor DNA] explode all trees | 68 |
| #8 | MeSH descriptor: [DNA Breaks] explode all trees | 31 |
| #9 | (liquid biopsy) OR (circulat* tumo?r cell*) OR (liquid* NEAR/3 biops*) OR (plasma NEAR/3 biops*) OR (plasma NEAR/3 DNA*) OR (urine NEAR/3 DNA*) OR (cerebrospinal fluid NEAR/3 DNA*) OR (pleural NEAR/3 DNA*) OR (peritoneal fluid NEAR/3 DNA*) OR (saliva NEAR/3 DNA*) OR (stool NEAR/3 DNA*) OR (circulating NEAR/3 (cell* or tumo?r* or neoplas*)) OR (circulat* NEAR/3 (cell* or tumo?r* or neoplas*) NEAR/2 DNA) OR (ctdna) OR (cell free nucleic acid) OR (circulating cell free rna or ccfrna) OR (circulating cell free dna or ccfdna) OR (biolog* marker*) OR ((cancer* or neoplasm*) NEAR/3 blood NEAR/3 test*) | 16094 |
| #10 | #4 OR #5 OR #6 OR #7 OR #8 OR #9 | 22102 |
| #11 | #3 and #10 | 12814 |
| #12 | MeSH descriptor: [Technology Assessment, Biomedical] explode all trees | 325 |
| #13 | MeSH descriptor: [Cost-Benefit Analysis] explode all trees | 9866 |
| #14 | MeSH descriptor: [Cost-Effectiveness Analysis] explode all trees | 29 |
| #15 | (cost* NEAR/3 analys* neoplas*) OR ((health* or boimed*) NEAR/2 technolog* NEAR/3 asses*) OR (economic* NEAR/3 evaluat*) OR (health* NEAR/3 economic*) OR (economic* NEAR/3 analys*) OR (cost effective*) | 57549 |
| #16 | #12 OR #13 OR #14 #15 | 10075 |
| #17 | #11 and #16 with Publication Year from 2001 to 2023, in Trials | 50 |
| **Web of Science** | | |
| 1 | TS= (cancer OR tumo?r OR neoplas* OR carcinoma*) | 3916334 |
| 2 | TS=((liquid biopsy) OR (circulat* tumo?r cell*) OR (liquid* NEAR/3 biops*) OR (plasma NEAR/3 biops*) OR (plasma NEAR/3 DNA*) OR (urine NEAR/3 DNA*) OR (cerebrospinal fluid NEAR/3 DNA*) OR (pleural NEAR/3 DNA*) OR (peritoneal fluid NEAR/3 DNA*) OR (saliva NEAR/3 DNA*) OR (stool NEAR/3 DNA*) OR (circulating NEAR/3 (cell* or tumo?r* or neoplas*)) OR (circulat* NEAR/3 (cell* or tumo?r* or neoplas*) NEAR/2 DNA) OR (ctdna) OR (cell free nucleic acid) OR (circulating cell free rna or ccfrna) OR (circulating cell free dna or ccfdna) OR (biolog* marker*) OR ((cancer* or neoplasm*) NEAR/3 blood NEAR/3 test*)) | 175685 |
| 3 | #2 AND #1 | 70712 |
| 4 | TS=((cost* NEAR/3 analys* neoplas*) OR ((health* or boimed*) NEAR/2 technolog* NEAR/3 asses*) OR (economic* NEAR/3 evaluat*) OR (health* NEAR/3 economic*) OR (economic* NEAR/3 analys*) OR (cost effective*) ) | 766760 |
| 5 | #4 AND #3 | 1001 |
| **Center for Review and Dissemination** | | |
| 1 | (cancer OR tumo?r OR neoplas* OR carcinoma*) | 14067 |
| 2 | (liquid biopsy) OR (circulat* tumo?r cell*) OR (liquid* NEAR/3 biops*) OR (plasma NEAR/3 biops*) OR (plasma NEAR/3 DNA*) OR (urine NEAR/3 DNA*) OR (cerebrospinal fluid NEAR/3 DNA*) OR (pleural NEAR/3 DNA*) OR (peritoneal fluid NEAR/3 DNA*) OR (saliva NEAR/3 DNA*) OR (stool NEAR/3 DNA*) | 26 |
| 3 | (ctdna) OR (cell free nucleic acid) OR (circulating cell free rna or ccfrna) OR (circulating cell free dna or ccfdna) OR (biolog* marker*) | 25 |
| 4 | #2 OR #3 | 51 |
| 5 | (cost* NEAR/3 analys* neoplas*) OR (health* NEAR/2 technolog* NEAR/3 asses*) OR (economic* NEAR/3 evaluat*) OR (health* NEAR/3 economic*) OR (economic* NEAR/3 analys*) OR (cost effective*) | 16678 |
| 6 | #4 AND #5 | 2 |
| 7 | (#4 AND #5) FROM 2001 TO 2023 | 1 |

**Table S2 Assessment of PRISMA 2020 checklist**

| **Topic** | **Item** | **Checklist Item** | **Number of page** |
| --- | --- | --- | --- |
| **TITLE** |  |  |  |
| **Title** | 1 | Identify the report as a systematic review. | Title page |
| **ABSTRACT** |  |  |  |
| **Abstract** | 2 | See the PRISMA 2020 for Abstracts checklist | 1 |
| **INTRODUCTION** |  |  |  |
| **Rationale** | 3 | Describe the rationale for the review in the context of existing knowledge. | 3 |
| **Objectives** | 4 | Provide an explicit statement of the objective(s) or question(s) the review addresses. | 3 |
| **METHODS** |  |  |  |
| **Eligibility criteria** | 5 | Specify the inclusion and exclusion criteria for the review and how studies were grouped for the syntheses. | 3,4 |
| **Information sources** | 6 | Specify all databases, registers, websites, organisations, reference lists and other sources searched or consulted to identify studies. Specify the date when each source was last searched or consulted. | 3,4 |
| **Search strategy** | 7 | Present the full search strategies for all databases, registers and websites, including any filters and limits used. | Table S1 |
| **Selection process** | 8 | Specify the methods used to decide whether a study met the inclusion criteria of the review, including how many reviewers screened each record and each report retrieved, whether they worked independently, and if applicable, details of automation tools used in the process. | 4 |
| **Data collection process** | 9 | Specify the methods used to collect data from reports, including how many reviewers collected data from each report, whether they worked independently, any processes for obtaining or confirming data from study investigators, and if applicable, details of automation tools used in the process. | 4 |
| **Data items** | 10a | List and define all outcomes for which data were sought. Specify whether all results that were compatible with each outcome domain in each study were sought (e.g. for all measures, time points, analyses), and if not, the methods used to decide which results to collect. | Table S1 |
|  | 10b | List and define all other variables for which data were sought (e.g. participant and intervention characteristics, funding sources). Describe any assumptions made about any missing or unclear information. | N/A |
| **Study risk of bias assessment** | 11 | Specify the methods used to assess risk of bias in the included studies, including details of the tool(s) used, how many reviewers assessed each study and whether they worked independently, and if applicable, details of automation tools used in the process. | Table S3 |
| **Effect measures** | 12 | Specify for each outcome the effect measure(s) (e.g. risk ratio, mean difference) used in the synthesis or presentation of results. | 4 |
| **Synthesis methods** | 13a | Describe the processes used to decide which studies were eligible for each synthesis (e.g. tabulating the study intervention characteristics and comparing against the planned groups for each synthesis (item 5)). | 4,5 |
|  | 13b | Describe any methods required to prepare the data for presentation or synthesis, such as handling of missing summary statistics, or data conversions. | 4,5 |
|  | 13c | Describe any methods used to tabulate or visually display results of individual studies and syntheses. | 4,5 |
|  | 13d | Describe any methods used to synthesize results and provide a rationale for the choice(s). If meta-analysis was performed, describe the model(s), method(s) to identify the presence and extent of statistical heterogeneity, and software package(s) used. | 4,5 |
|  | 13e | Describe any methods used to explore possible causes of heterogeneity among study results (e.g. subgroup analysis, meta-regression). | N/A |
|  | 13f | Describe any sensitivity analyses conducted to assess robustness of the synthesized results. | N/A |
| **Reporting bias assessment** | 14 | Describe any methods used to assess risk of bias due to missing results in a synthesis (arising from reporting biases). | N/A |
| **Certainty assessment** | 15 | Describe any methods used to assess certainty (or confidence) in the body of evidence for an outcome. | N/A |
| **RESULTS** |  |  |  |
| **Study selection** | 16a | Describe the results of the search and selection process, from the number of records identified in the search to the number of studies included in the review, ideally using a flow diagram | 5 |
|  | 16b | Cite studies that might appear to meet the inclusion criteria, but which were excluded, and explain why they were excluded. | N/A |
| **Study characteristics** | 17 | Cite each included study and present its characteristics. | 5 |
| **Risk of bias in studies** | 18 | Present assessments of risk of bias for each included study. | Table S4 |
| **Results of individual studies** | 19 | For all outcomes, present, for each study: (a) summary statistics for each group (where appropriate) and (b) an effect estimate and its precision (e.g. confidence/credible interval), ideally using structured tables or plots. | 5 |
| **Results of syntheses** | 20a | For each synthesis, briefly summarise the characteristics and risk of bias among contributing studies. | 5,6,7 |
|  | 20b | Present results of all statistical syntheses conducted. If meta-analysis was done, present for each the summary estimate and its precision (e.g. confidence/credible interval) and measures of statistical heterogeneity. If comparing groups, describe the direction of the effect. | 5,6,7 |
|  | 20c | Present results of all investigations of possible causes of heterogeneity among study results. | 5,6,7 |
|  | 20d | Present results of all sensitivity analyses conducted to assess the robustness of the synthesized results. | 5,6,7 |
| **Reporting biases** | 21 | Present assessments of risk of bias due to missing results (arising from reporting biases) for each synthesis assessed. | N/A |
| **Certainty of evidence** | 22 | Present assessments of certainty (or confidence) in the body of evidence for each outcome assessed. | N/A |
| **DISCUSSION** |  |  |  |
| **Discussion** | 23a | Provide a general interpretation of the results in the context of other evidence. | 8 |
|  | 23b | Discuss any limitations of the evidence included in the review. | 9 |
|  | 23c | Discuss any limitations of the review processes used. | 9 |
|  | 23d | Discuss implications of the results for practice, policy, and future research. | 8,9 |
| **OTHER INFORMATION** |  |  |  |
| **Registration and protocol** | 24a | Provide registration information for the review, including register name and registration number, or state that the review was not registered. | 3 |
|  | 24b | Indicate where the review protocol can be accessed, or state that a protocol was not prepared. | N/A |
|  | 24c | Describe and explain any amendments to information provided at registration or in the protocol. | N/A |
| **Support** | 25 | Describe sources of financial or non-financial support for the review, and the role of the funders or sponsors in the review. | 9,10 |
| **Competing interests** | 26 | Declare any competing interests of review authors. | 10 |
| **Availability of data, code and other materials** | 27 | Report which of the following are publicly available and where they can be found: template data collection forms; data extracted from included studies; data used for all analyses; analytic code; any other materials used in the review. | 10 |

# **Table S3 Items and requirement of CHEERS Checklist 2022**

| **Item** | **Requirements** |
| --- | --- |
| 1 | Identify the study as an economic evaluation and specify the interventions being compared. |
| 2 | Provide a structured summary that highlights context, key methods, results and alternative analyses. |
| 3 | Give the context for the study, the study question and its practical relevance for decision making in policy or practice. |
| 4 | Indicate whether a health economic analysis plan was developed and  where available. |
| 5 | Describe characteristics of the study population (such as age range, demographics, socioeconomic, or clinical characteristics). |
| 6 | Provide relevant contextual information that may influence findings. |
| 7 | Describe the interventions or strategies being compared and why chosen. |
| 8 | State the perspective(s) adopted by the study and why chosen. |
| 9 | State the time horizon for the study and why appropriate. |
| 10 | Report the discount rate(s) and reason chosen. |
| 11 | Describe what outcomes were used as the measure(s) of benefit(s) and harm(s). |
| 12 | Describe how outcomes used to capture benefit(s) and harm(s) were measured. |
| 13 | Describe the population and methods used to measure and value outcomes. |
| 14 | Describe how costs were valued. |
| 15 | Report the dates of the estimated resource quantities and unit costs, plus the currency and year of conversion. |
| 16 | If modelling is used, describe in detail and why used. Report if the model  is publicly available and where it can be accessed. |
| 17 | Describe any methods for analysing or statistically transforming data, any extrapolation methods, and approaches for validating any model used. |
| 18 | Describe any methods used for estimating how the results of the study vary for sub-groups. |
| 19 | Describe how impacts are distributed across different individuals  or adjustments made to reflect priority populations. |
| 20 | Describe methods to characterize any sources of uncertainty in the analysis. |
| 21 | Describe any approaches to engage patients or service recipients, the general public, communities, or stakeholders (e.g., clinicians or payers) in the design of the study. |
| 22 | Report all analytic inputs (e.g., values, ranges, references) including uncertainty or distributional assumptions. |
| 23 | Report the mean values for the main categories of costs and outcomes of interest and summarise them in the most appropriate overall measure. |
| 24 | Describe how uncertainty about analytic judgments, inputs, or projections  affect findings. Report the effect of choice of discount rate and time horizon, if applicable. |
| 25 | Report on any difference patient/service recipient, general public, community, or stakeholder involvement made to the approach or findings of the study |
| 26 | Report key findings, limitations, ethical or equity considerations not captured, and how these could impact patients, policy, or practice. |
| 27 | Describe how the study was funded and any role of the funder in the identification, design, conduct, and reporting of the analysis |
| 28 | Report authors conflicts of interest according to journal or  International Committee of Medical Journal Editors requirements. |

# **Table S4 CHEERS checklist completeness assessment of included studies**

| Item label | Harris 2019[1] | Miller 2021[2] | Poort 2022[3] | Ladabaum 2014[4] | Ladabaum 2013[5] | Karlitz 2022[6] | Redwood 2021[7] | Fisher 2021[8] | Hathway 2020[9] | Naber 2019[10] | Ladabaum 2016[11] | Kingsley 2016[12] | Lansdorp-Vogelaar 2010 [13] | Wu 2006[14] | Peterse 2021[15] | Lew 2018[16] | Benamouzig 2021[17] | Barré 2020[18] | Reports fulfilled the item (%) |
| --- | --- | --- | --- | --- | --- | --- | --- | --- | --- | --- | --- | --- | --- | --- | --- | --- | --- | --- | --- |
| Title | R | R | R | R | R | R | R | R | R | R | R | R | R | R | R | R | R | R | 100% |
| Abstract | R | R | R | R | R | R | R | R | R | R | R | R | R | R | R | R | R | R | 100% |
| Background & Objectives | R | R | R | R | R | R | R | R | R | R | R | R | R | R | R | R | R | R | 100% |
| Health economic analysis plan | NR | NR | NR | NR | NR | NR | R | NR | NR | NR | NR | NR | NR | NR | NR | R | NR | NR | 11% |
| Study population | R | R | R | R | R | R | R | R | R | R | R | R | R | R | R | R | R | R | 100% |
| Setting and location | R | PR | R | R | R | R | R | R | R | R | R | R | R | R | R | R | R | R | 97% |
| Comparator(s) | PR | R | R | R | R | R | R | R | R | R | R | R | R | R | R | R | R | R | 97% |
| Perspective | R | R | R | R | R | R | R | R | R | R | R | R | R | R | R | R | R | R | 100% |
| Time horizon | R | R | R | PR | PR | R | PR | PR | R | PR | R | PR | PR | PR | PR | PR | R | PR | 69% |
| Discount rate | R | R | R | R | R | R | R | R | R | NR | R | R | R | R | R | R | R | R | 94% |
| Selection of outcomes | R | R | R | R | R | R | R | R | R | R | R | R | R | R | R | R | R | R | 100% |
| Measurement of outcomes | R | R | PR | PR | PR | R | R | R | R | R | R | R | R | R | PR | PR | PR | PR | 81% |
| Valuation of outcomes | R | PR | PR | PR | PR | R | R | R | R | PR | R | PR | PR | R | PR | PR | PR | PR | 69% |
| Measurement and valuation of resources and costs | PR | PR | PR | PR | PR | PR | PR | PR | PR | PR | PR | PR | PR | PR | PR | PR | PR | PR | 50% |
| Currency, price date, and conversion | PR | PR | PR | PR | PR | PR | PR | PR | PR | PR | PR | PR | PR | PR | PR | PR | PR | PR | 50% |
| Rationale and description of model | PR | PR | PR | PR | PR | PR | PR | PR | PR | PR | PR | R | PR | R | PR | R | PR | PR | 58% |
| Analytics and assumptions | PR | PR | PR | PR | PR | PR | PR | R | PR | PR | R | PR | PR | R | PR | PR | PR | PR | 58% |
| Characterizing heterogeneity | R | R | R | R | R | R | R | R | R | R | R | R | R | R | R | R | R | R | 100% |
| Characterizing distributional effects | NR | NR | NR | NR | NR | NR | NR | NR | NR | NR | NR | NR | NR | NR | NR | NR | NR | NR | 0% |
| Characterizing uncertainty | R | R | R | R | R | R | R | R | R | R | R | R | R | R | R | R | R | R | 100% |
| Approach to engagement with patients and others affected by the study | NR | NR | NR | NR | NR | NR | NR | NR | NR | NR | NR | NR | NR | NR | NR | NR | NR | NR | 0% |
| Study parameters | PR | R | PR | PR | R | R | R | R | R | R | R | R | PR | R | R | R | R | PR | 83% |
| Summary of main results | R | R | R | R | R | R | R | R | R | R | R | R | R | R | R | R | R | R | 100% |
| Effect of uncertainty | R | R | R | R | R | R | R | R | R | R | R | R | R | R | R | R | R | R | 100% |
| Effect of engagement with patients and others affected by the study | NR | NR | NR | NR | NR | NR | NR | NR | NR | NR | NR | NR | NR | NR | NR | NR | NR | NR | 0% |
| Study findings, limitations, generalizability, and current knowledge | R | R | R | R | R | R | R | R | R | R | R | R | R | R | R | R | R | R | 100% |
| Source of funding | R | R | R | R | R | R | R | R | R | R | R | R | R | R | R | R | R | R | 100% |
| Conflicts of interest | R | R | R | R | R | R | R | R | R | R | R | R | R | R | R | R | R | R | 100% |
| Total score | 21 | 20.5 | 20.5 | 20 | 20.5 | 22 | 22.5 | 22 | 22 | 20 | 22.5 | 21.5 | 20.5 | 22.5 | 20.5 | 22 | 21 | 20 | - |
| Item fulfilled (%) | 75% | 73% | 73% | 71% | 73% | 79% | 80% | 79% | 79% | 71% | 80% | 77% | 73% | 80% | 73% | 79% | 75% | 71% | - |
| Quality category | very good | very good | very good | very good | very good | very good | very good | very good | very good | very good | very good | very good | very good | very good | very good | very good | very good | very good | - |

R: Reported (score 1); NR: Not Reported (score 0); P: Partially Reported (score 0.5); Quality categories: Excellent scoring, >85.0%; very good scoring, 70.0%–84.0%; good scoring, 55.0%–69.0%; insufficient scoring, <55.0%.

**Reference**

1. Harris J, Saraswathula A, Kaplun B, Qian Y, Chan K, Chan A et al: Cost-effectiveness of Screening for Nasopharyngeal Carcinoma among Asian American Men in the United States.Otolaryngol Head Neck Surg*.*2019;161(1):82-90.

2. Miller JA, Le Q-T, Pinsky BA, Wang H: Cost-Effectiveness of Nasopharyngeal Carcinoma Screening With Epstein-Barr Virus Polymerase Chain Reaction or Serology in High-Incidence Populations Worldwide.JNCI*.*2021;113(7):852-862.

3. van der Poort E, van Ravesteyn N, van den Broek J, de Koning H: The Early Detection of Breast Cancer Using Liquid Biopsies: Model Estimates of the Benefits, Harms, and Costs.Cancers*.*2022;14(12):2951.

4. Ladabaum U, Alvarez-Osorio L, Rösch T, Brueggenjuergen B: Cost-effectiveness of colorectal cancer screening in Germany: current endoscopic and fecal testing strategies versus plasma methylated Septin 9 DNA.Endosc Int Open*.*2014;2(2):E96-E104.

5. Ladabaum U, Allen J, Wandell M, Ramsey S: Colorectal cancer screening with blood-based biomarkers: cost-effectiveness of methylated septin 9 DNA versus current strategies.Cancer Epidemiol Biomarkers Prev 2013;22(9):1567-1576.

6. Karlitz J, Fendrick A, Bhatt J, Coronado G, Jeyakumar S, Smith N et al: Cost-Effectiveness of Outreach Strategies for Stool-Based Colorectal Cancer Screening in a Medicaid Population.Popul Health Manage*.*2022;25(3):343-351.

7. Redwood DG, Dinh TA, Kisiel JB, Borah BJ, Moriarty JP, Provost EM et al: Cost-Effectiveness of Multitarget Stool DNA Testing vs Colonoscopy or Fecal Immunochemical Testing for Colorectal Cancer Screening in Alaska Native People.Mayo Clin Proc*.*2021;96(5):1203-1217.

8. Fisher D, Karlitz J, Jeyakumar S, Smith N, Limburg P, Lieberman D et al: Real-world cost-effectiveness of stool-based colorectal cancer screening in a Medicare population.J Med Econ*.*2021;24(1):654-664.

9. Hathway JM, Miller-Wilson L-A, Jensen IS, Ozbay B, Regan C, Jena AB et al: Projecting total costs and health consequences of increasing mt-sDNA utilization for colorectal cancer screening from the payer and integrated delivery network perspectives.J Med Econ*.*2020;23(6):581-592.

10. Naber SK, Knudsen AB, Zauber AG, Rutter CM, Fischer SE, Pabiniak CJ et al: Cost-effectiveness of a multitarget stool DNA test for colorectal cancer screening of Medicare beneficiaries.PLoS One*.*2019;14(9):e0220234.

11. Ladabaum U, Mannalithara A: Comparative Effectiveness and Cost Effectiveness of a Multitarget Stool DNA Test to Screen for Colorectal Neoplasia.Gastroenterology*.*2016;151(3):427-439.e426.

12. Kingsley J, Karanth S, Revere F, Agrawal D: Cost Effectiveness of Screening Colonoscopy Depends on Adequate Bowel Preparation Rates - A Modeling Study.PLoS One*.*2016;11(12):e0167452.

13. Lansdorp-Vogelaar I, Kuntz K, Knudsen A, Wilschut J, Zauber A, van Ballegooijen M: Stool DNA Testing to Screen for Colorectal Cancer in the Medicare Population A Cost-Effectiveness Analysis.Ann Intern Med*.*2010;153(6):368-377.

14. Wu G, Wang Y, Yen A, Wong J, Lai H, Warwick J et al: Cost-effectiveness analysis of colorectal cancer screening with stool DNA testing in intermediate-incidence countries.BMC Cancer 2006;6:136.

15. Peterse E, Meester R, de Jonge L, Omidvari A, Alarid-Escudero F, Knudsen A et al: Comparing the Cost-Effectiveness of Innovative Colorectal Cancer Screening Tests.JNCI*.*2021;113(2):154-161.

16. Lew J, St John D, Macrae F, Emery J, Ee H, Jenkins M et al: Evaluation of the benefits, harms and cost-effectiveness of potential alternatives to iFOBT testing for colorectal cancer screening in Australia.Int J Cancer*.*2018;143(2):269-282.

17. Benamouzig R, Barré S, Saurin J, Leleu H, Vimont A, Taleb S et al: Cost-effectiveness analysis of alternative colorectal cancer screening strategies in high-risk individuals.Therap Adv Gastroenterol*.*2021;14:17562848211002359.

18. Barré S, Leleu H, Benamouzig R, Saurin J, Vimont A, Taleb S et al: Cost-effectiveness analysis of alternative colon cancer screening strategies in the context of the French national screening program.Therap Adv Gastroenterol*.*2020;13:1756284820953364.
